# *Acanthus ilicifolius* Methanolic Extract for Oral Candidiasis Treatment through Tongue Epithelial STAT3 and Cell Death Evaluation

**DOI:** 10.1055/s-0042-1760298

**Published:** 2023-02-10

**Authors:** Syamsulina Revianti, Dwi Andriani, Agni Febrina Pargaputri, Mardiyanto Riski Hartono

**Affiliations:** 1 Department of Oral Biology, Faculty of Dentistry, Universitas Hang Tuah, Surabaya, Surabaya, Indonesia

**Keywords:** cell death, STAT3, immunosuppression, oral candidiasis, corticosteroids, antifungals

## Abstract

**Objectives** This study aimed to evaluate the effects of topical application of *Acanthus ilicifolius* methanolic extract on signal transducer and activator of transcription 3 (STAT3) expression and tongue epithelial cell death caused by oral candidiasis under immunosuppressive conditions.

**Materials and Methods** To obtain the oral candidiasis model, 20 healthy male *Rattus norvegicus* (Wistar) rats were administered oral dexamethasone and tetracycline for 14 days and oral candidiasis was induced with *Candida albicans* (ATCC-10231) 1 McFarland. They were then randomized into four groups—immunosuppression (K-), oral candidiasis (K + ), nystatin treatment (P1), and 20% *A. ilicifolius* methanolic extract treatment (P2) and were treated for 14 days. Histological analyses of cell death and candida invasion and immunohistochemical analysis of STAT3 in epithelial cells were performed.

**Statistical Analysis** Epithelial cell death data were analyzed using one-way analysis of variance (ANOVA) and the post hoc Games–Howell test ( *p* < 0.05) and STAT3 expression with one-way ANOVA and the post hoc least significant difference test ( *p* < 0.05).

**Results** Cell death was significantly different between K- and K+ and between K+ and P1 and P2 ( *p* < 0.05); there were no significant differences between K- and P1 and P2 and between P1 and P2 ( *p* > 0.05). STAT3 expression was significantly different between K- and P1 and P2 and between K+ and P1 and P2 ( *p* < 0.05), but there were no significant differences between K+ and K- and between P1 and P2 ( *p* > 0.05).

**Conclusion** Topical administration of *A. ilicifolius* methanol extract increased STAT3 expression and decreased tongue epithelial cell death caused by oral candidiasis.

## Introduction

Indonesia is rich in biodiversity 1 and is surrounded by a vast ocean with abundant marine species. It also has one of the richest stores of medicinal plants in the world, which are used in herbal medicine and are known to have few side effects. *Acanthus ilicifolius* L., found in the coastal areas of Indonesia, has antifungal and anti-inflammatory properties due to its chemical components, including flavonoids, alkaloids, glycosides, polyphenols, tannins, and steroids. 2 Methanolic extracts of *A. ilicifolius* are potent inhibitors of *Candida albicans* growth and show anti-inflammatory effects via the mitogen-activated protein kinase pathway. 3 These extracts have strong antioxidant and antifungal activity, and the plant's phytochemical compounds are potential candidates for antifungal therapy. 2

*C. albicans* , the main causative agent of oral yeast infection, is a commensal organism that transforms into a pathogen in hosts suffering from systemic diseases, such as diabetes mellitus, leukopenia, human immunodeficiency virus/acquired immunodeficiency syndrome (AIDS), and malignancies, as well as conditions related to hematinic deficiency, carbohydrate-rich diets, drugs, or immunosuppression (e.g., autosomal dominant hyper-immunoglobulin E syndrome [AD-HIES]). 4 5 6 Candida is a frequent invader of the oral mucosa. The host immune response in the oral cavity is oriented toward a more tolerogenic state and, therefore, local innate immune defenses play a role in preventing candida invasion of the host. In particular, apart from being able to prevent candida adherence to epithelial cells, saliva is enriched with anti-candida peptides, which are considered part of the host's innate immunity. 4 AD-HIES is characterized by impaired production of Th17 cells and Th17-derived cytokines caused by the signal transducer and activator of transcription 3 (STAT3) autosomal dominant mutation. HIES patients have low salivary concentrations of critical candidacidal peptides, including histatins and β-defensin 2 (BD2), which are activated by interleukin (IL)-17. 6 This condition can cause *C. albicans* to become pathogenic and more easily invade the mucosa.

HIES is most often due to mutations in the STAT3 gene. STAT3 is one of the major signaling molecules of the IL-23 receptor 7 and is a key transcription factor downstream of cytokine signaling of IL-6, IL-21, IL-10, and IL-23, among other immune mediators. STAT3 has also been defined as a key transcription factor in Th17 differentiation. 8 The IL-17/Th17 response is particularly relevant for maintaining the barrier integrity of the host mucosa and preventing infection by *C. albicans* . 9 Patients and mice with defects in Th17/IL-17 immunity have been reported to have severe susceptibility to oral yeast infections. 8 The importance of STAT3 in Th17 regulation for the mucosal defense of *C. albicans* is warranted to be investigated.

During infection, interactions between oral mucosal epithelial cells and *C. albicans* result in epithelial cell death. *C. albicans* induces early apoptotic changes in oral epithelial cells. 10 Moyes et al found that the dynamics of cell death during *C. albicans* infection of epithelial cells involves early induction of apoptosis, followed by necrosis. 11 This causes the development of mucosal ulceration generally associated with discomfort in patients with clinical manifestations of oral candidiasis. 10 Based on the description above, we studied the effects of *A. ilicifolius* therapy on oral candidiasis under immunosuppressed conditions, with the aim of understanding the action of the herbal extract through the STAT3 pathway, which is one of the body's defense lines against fungal invasion.

## Materials and Methods

### Research Methods

This study was a laboratory experiment with a posttest-only control group design. Our research subjects were male Wistar strain *Rattus norvegicus* rats, weighing approximately 200 g. The rats were randomly divided into four groups with five replicates each: K-, immunosuppressed rat group (control); K + , immunosuppressed rat group with oral candidiasis; P1, immunosuppressed rat group with oral candidiasis treated with nystatin; and P2, immunosuppressed rat group with oral candidiasis treated with *A. ilicifolius* methanolic extract (20%). We used a simple random sampling technique. The study was evaluated for feasibility by the Research Ethics Committee of the Faculty of Dentistry, Hang Tuah University (EC/016/KEPK-FKGUHT/VIII/2021).

### Extract Preparation

Methanol extraction of *A. ilicifolius* leaves was based on Andriani et al and used a maceration technique. *A. ilicifolius* leaves were dried in an oven at 45°C and then ground into a coarse powder. The powder was immersed in 96% methanol for 48 hours in an Erlenmeyer flask lined with aluminum foil, with occasional shaking and stirring. The mix was then filtered with Whatman Grade 1 filter paper. The solvent was evaporated at low pressure using a rotary evaporator to obtain a viscous mass, which was evaporated again in a water bath to separate the extract from the solvent to produce pure *A. ilicifolius* leaf extract. 12

### Treatment of Experimental Animals

#### Induction of Immunosuppressed Oral Candidiasis

To induce immunosuppression in mice, we treated them orally with dexamethasone (Dexamethasone, PT. Phapros tbk., Indonesia) 0.5 mg/day and tetracycline (Tetracycline, PT Novapharin, Indonesia) 1%/day for 7 days. On day 8, the dexamethasone dose was reduced by 10%, while the tetracycline dose remained at 1%. Between day 8 and day 20, the rat tongue was inoculated with 0.5 mL *C. albicans* ATCC 10231 three times a week for 2 weeks to induce oral candidiasis; McFarland 1 (3 × 108 CFU/mL). 13 14

#### Examination of Hyphae and Yeast

Hyphae were examined after staining with periodic acid–Schiff staining (Sigma, 395B, Sigma-Aldrich Pte Ltd, United States). Observations of the presence of yeast or hyphae on the upper epithelium were made with a light microscope (Olympus CX-31) supported by an Optilab Viewer at 400× magnification.

#### Oral Candidiasis Treatment

In the P1 group immunosuppressed rats with oral candidiasis treated with only nystatin (Nystatin, PT. Lapi Laboratories, Indonesia), and in P2 immunosuppressed rats with oral candidiasis treated with *A. ilicifolius* methanolic extract (20%). Nystatin (alone) and *A. ilicifolius* methanolic extract (20%) were applied to the dorsum tongue of rats twice a day for 2 weeks. Each rat was then sacrificed, and the tongue was biopsied. 13 14

#### Cell Death Measurement

Cell death was determined following staining with hematoxylin and eosin (Indo Reagen, AKD 10204600180, Indonesia). Measurement was made using a light microscope (Olympus CX-31) supported with an Optilab Viewer at 1,000× magnification. Cell death was measured by counting dead cells (pyknotic cell nuclei, karyorrhexis, and karyolysis) from the stratum corneum to the stratum basale of the epithelium. We combined the measurements of 10 selected fields of view using raster image software, and then calculated the average value.

#### STAT3 Measurement

STAT3 expression was measured with immunohistological staining (Santa Cruz Biotechnology, Santa Cruz, California, United States). The measurement was performed using a light microscope (Olympus CX-31) supported with an Optilab Viewer at 1,000× magnification. STAT3 expression was examined by counting the presence of brown precipitates on the epithelial cell in 10 selected fields of view provided by raster image software, and then the average value was calculated for each group.

## Results

**Fig. 1:**
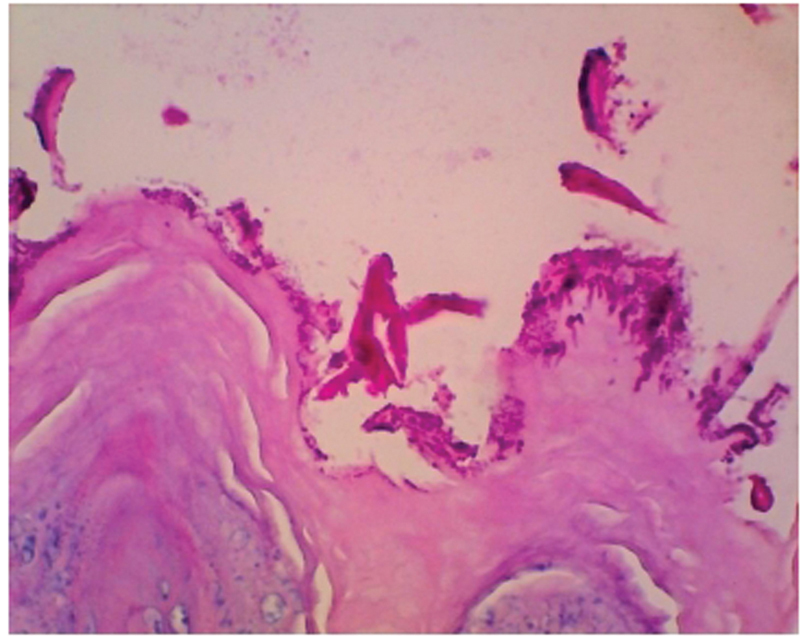
Hyphae invasion in the epithelial site of rat's tongue (periodic acid–Schiff [PAS] staining, 400× magnification).

Invasion of rats' tongues by *C. albicans ATCC-10231* hyphae under immunosuppression is shown in Fig. 1 . Hyphal invasion of the upper layer of the epithelium can be observed, which indicates infection by *C. albicans* .

**Fig. 2:**
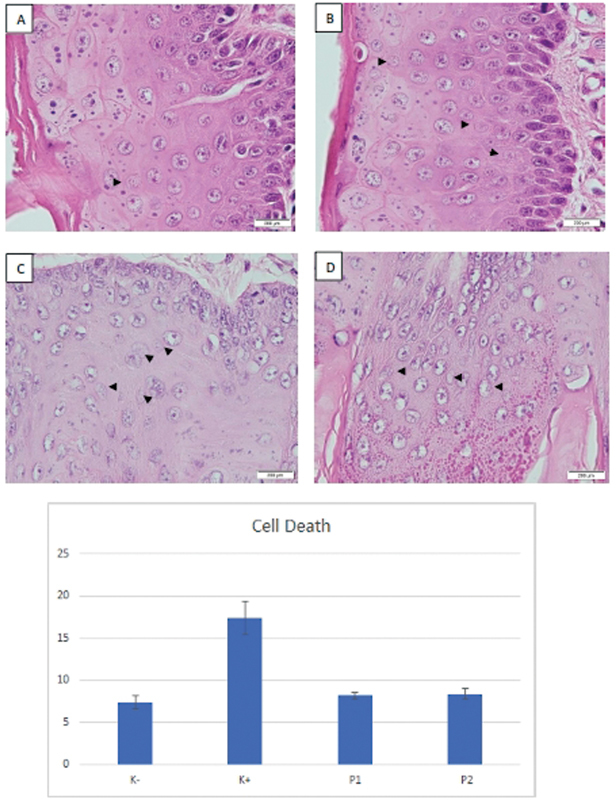
Cell death on rat's tongue epithelium pyknotic cell nuclei, karyolysis, and karyolysis (arrowhead). ( **A** ) Immunosuppression rats group (K-); ( **B** ) oral candidiasis immunosuppressed rats group (K + ); ( **C** ) oral candidiasis immunosuppressed rats group treated with nystatin (P1); and ( **D** ) oral candidiasis immunosuppressed rats group treated with *Acanthus ilicifolius* methanolic extract 20% (P2) (hematoxylin and eosin [H&E] staining, 1,000× magnification).

Morphologically, dead cells show pyknotic cell nuclei, karyolysis, and karyolysis ( Fig. 2 ). The number of cell death in the epithelium is shown in table 1 . The result show, there was significantly different between K− and K+ and between K+ and P1 and P2 ( *p* < 0.05) and there were no significant differences between K− and P1 and P2 and between P1 and P2 ( *p* > 0.05).

**Fig. 3:**
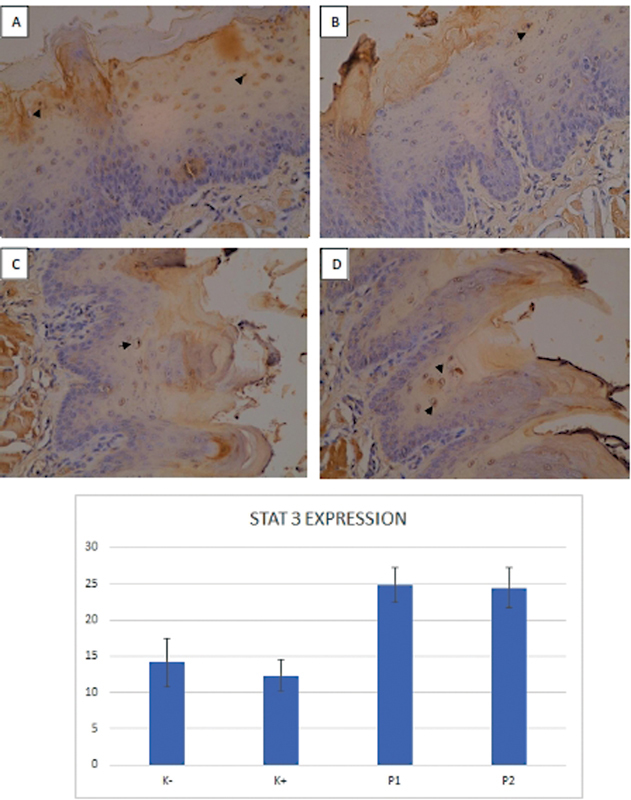
Signal transducer and activator of transcription 3 (STAT3) expression in the epithelial site of rat's tongues (arrowhead). ( **A** ) Immunosuppression rats' group (K−); ( **B** ) oral candidiasis immunosuppressed rats' group (K Þ); ( **C** ) oral candidiasis immunosuppressed rats group treated with nystatin (P1); and ( **D** ) oral candidiasis immunosuppressed rats group treated with Acanthus ilicifolius methanolic extract 20% (P2) (immunohistochemistry staining, 400 magnification).

An overview of STAT3 expression in this study is shown in Figure 3 and table 2 . STAT3 expression was significantly different between K− and P1 and P2 and between K+ and P1 and P2 ( *p* < 0.05), but there were no significant differences between K+ and K− and between P1 and P2 ( *p* > 0.05).

## Discussion

*C. albicans* is an opportunistic fungal pathogen that can cause widespread infections. It forms pseudohyphae after it adheres to and grows on epithelial cells. *C. albicans* hyphae formation indicates the process of pathogen invasion of the body. 11 Hyphae of *C. albicans* secrete a toxin, candidalysin, which plays an important role in destroying epithelial cells. Candidalysin causes damage to epithelial cells by inducing necrosis, which increases with increasing concentration of the toxin. 15 Andriani et al reported the presence of *C. albicans* biofilm on the tongue epithelium of rats under immunosuppressed conditions. 16 Based on Andriani et al, we performed a fungal induction treatment on the tongue. Bachtiar et al reported high expression of hypha-specific genes, ALS3 and HWP1, on the dorsal surface of the tongue of patients wearing dentures. These two genes are associated with biofilm in fungal infections. 17

In this study, the mean cell death (pyknotic cell nuclei, karyorrhexis, and karyolysis in the epithelial cells) of the immunosuppressed group was significantly lower than that of the immunosuppressed rats with oral candidiasis ( Table 1 ). This is because candida infection causes cell death by inducing apoptosis in epithelial cells and inhibiting antiapoptotic proteins, such as BD2 and Bcl XL, in macrophages and neutrophils. 11 Increased candidalysin concentration causes greater epithelial cell necrosis by attacking mitochondrial function, which leads to decreased metabolic activity and adenosine triphosphate and increased intercellular reactive oxygen species, and the induction of necrosis. 15

Cell death in the immunosuppressed group compared with that of the immunosuppressed group with oral candidiasis treated with nystatin or 20% *A. ilicifolius* extract was found to be lower on average but not significantly. Cell death in the immunosuppressed group with oral candidiasis was significantly higher than in the therapeutically treated groups. This shows that the therapy given was able to reduce infection by *C. albicans* . Nystatin is commonly used against fungal infections and plays an important role in the prophylaxis of oral and systemic candidiasis in full-term and preterm newborns, infants, and immunocompromised patients (e.g., patients with AIDS or cancer and organ transplant recipients), as it is associated with low incidence of drug interactions. 18 Previous studies have shown that *A. ilicifolius* extract has antifungal and antioxidant effects *in vitro* and antifungal activity in mice suffering from oral candidiasis under immunosuppressed conditions. 12 13 14

Cell death was not significantly different between the nystatin and 20% *A. ilicifolius* extract treatment groups. This shows that *A. ilicifolius* therapy has the same efficacy as nystatin. The methanolic extract of *A. ilicifolius* has antifungal activity and the best activity at a concentration of 20%. 12 This extract contains flavonoids, tannins, steroids, saponins, terpenoids, glycosides, and polyphenols. 2 19 2-Benzoxazolinone and benzoxazinoids in this extract show antifungal and antioxidant activity. 20 These secondary metabolites of *A. ilicifolius* extract contribute to its antifungal property by increasing the expression of Toll-like receptor 2, IL-22, and P38 in immunosuppressed rats with oral candidiasis. 3 13 14 These three markers are known to play a role in the inflammatory mechanism against *C. albicans* infection.

The STAT3 cascade plays an essential role in the inflammatory signaling pathway that modulates immune responses and has targeted genes which include inflammatory mediators, adhesion molecules, and cytokines. It has been shown that the severity of inflammation can lead to the occurrence of cell death. In general, this pathway transduces signals from activated receptors or intracellular kinases to the nucleus, therefore gene transcription would be activated and regulated. STAT3 proteins are stimulated and activated by cytokines. Considering that candidiasis is caused by the production and release of proinflammatory cytokines, the STAT3 pathway becomes an essential regulator of inflammation and cell death in the oral epithelial when exposed to an inflammatory condition like immunosuppression and oral candidiasis. 21

In this study, we found that nystatin and 20% *A. ilicifolius* extract could increase STAT3 expression and may lead to the production of Th17. STAT3 is an important transcription factor in several steps along the Th17 developmental pathway. Previous studies have shown that STAT3/Th17 plays an important role in oral antifungal defense, especially against *C. albicans* . 8 In this study, the immunosuppressed group with oral candidiasis and the immunosuppressed group had the least amount of STAT3. The level of STAT3 in these two groups was not significantly different, whereas it was significantly different from the levels in the groups treated with nystatin and 20% *A. ilicifolius* extract. This shows that under immunosuppressed and immunosuppressed as well as oral candidiasis conditions, the amount of STAT3 was reduced at a similar rate. This decrease resulted in a decrease in the body's ability to eliminate *C. albicans* . An experimental model with defects in STAT3/Th17 showed *C. albicans* overgrowth, and the bacterial community became dysbiotic with streptococci predominating. Investigations of a population of patients with genetic defects in STAT3 demonstrated the important role of STAT3-mediated immunity in oral mucosa infected with *C. albicans* and in the formation of oral commensals and fungi. 8

STAT3 expression significantly increased in the nystatin-treated group compared with the immunosuppressed control group and the immunosuppressed group with oral candidiasis. This shows that therapy using nystatin as an antifungal against candida infection is successful. Nystatin remains the first choice for oral candidiasis. Topical nystatin acts locally, and its administration works by direct contact with the affected tissue at a sufficient dose and over sufficient time. 5

STAT3 expression also increased significantly in the group treated with 20% *A. ilicifolius* extract compared with the immunosuppressed control group and the immunosuppressed group with oral candidiasis. This increase in expression was not significantly different from that due to nystatin therapy, suggesting that the administration of 20% *A. ilicifolius* extract can increase STAT3 to the same extent as nystatin therapy. STAT3 is a key transcription factor in Th17 differentiation. 8 Therefore, this increase can lead to an increase in Th17. Th17 plays an important role in the defense against *C. albicans* . The results of this study are also in line with those of previous studies that showed that topical therapy with 20% *A. ilicifolius* extract is effective against oral candidiasis under immunosuppression. 12 13 14

## Conclusion

The results of this study show that topical administration of *A. ilicifolius* methanolic extract leads to increased STAT3 expression and decreased tongue epithelial cell death in oral candidiasis under immunosuppressive conditions and that this effect is similar to that of nystatin. Further study was needed to investigate the correlation between the invasion of candida albicans, STAT3 expression, Th-17/IL-17, and the amount of epithelial cell death in oral candidiasis immunosuppressed rats to determine the severity of the disease.

**Table 1:** Mean ± standard deviation of cell death on the rat's tongue in each group

Group	Mean	
	Cell death	Standard deviation
K-	7.4 ^a^	0.812
K+	17.4 ^b^	1.965
P1	8.2 ^a^	0.37
P2	8.4 ^a^	0.6

Note: Superscripts with different small letters indicate statistically significance difference within the same column.

**Table 2:** Mean ± standard deviation of STAT3 expression on the rat's tongue in each group

Group	Mean	
	STAT3 expression	Standard deviation
K-	14.15 ^a^	3.37
K+	12.35 ^a^	2.12
P1	24.775 ^b^	2.39
P2	24.45 ^b^	2.78

Abbreviation: STAT3, signal transducer and activator of transcription 3.

Note: Superscripts with different small letters indicate statistically significance difference within the same column.
